# Medications for Hypertension Change the Secretome Profile from Marrow Stromal Cells and Peripheral Blood Monocytes

**DOI:** 10.1155/2020/8894168

**Published:** 2020-08-01

**Authors:** Nikunj Satani, Kaavya Giridhar, Chunyan Cai, Natalia Wewior, Dominique D. Norris, Jaroslaw Aronowski, Sean I. Savitz

**Affiliations:** ^1^Institute for Stroke and Cerebrovascular Diseases, McGovern Medical School at UTHealth, Houston, Texas, USA; ^2^Center for Clinical and Translational Sciences, McGovern Medical School at UTHealth, Houston, Texas, USA

## Abstract

Marrow stromal cells (MSCs) are in different stages of clinical trials for stroke patients. MSCs are proposed to promote recovery through the release of secretomes that modulate the function of beneficial immune cells. The majority of stroke patients have comorbidities including hypertension, for which they are prescribed antihypertensive medications that might affect the function of MSCs, when they are administered in stroke patients. Here, we studied the effects of common antihypertensive medications on the secretomes of human MSCs and their modulation of human monocytes (Mo) derived from stroke patients. MTT assay was used to assess the proliferation of MSCs after they were exposed to increased levels of antihypertensive medications. MSCs were exposed to the following medications: atenolol, captopril, and losartan. Monocytes were isolated from stroke patients with NIHSS ranging from 11 to 20 and from healthy controls. MSC-Mo cocultures were established, and a secretome profile was analyzed using the Magpix Multiplex cytokine array from Luminex technology. The linear mixed-effect model was used for statistical analysis. All analyses were performed using SAS 9.4, and *p* values less than 0.05 were considered significant. At clinically relevant levels, there was no change in MSC proliferation after exposure to atenolol, captopril, or losartan. Atenolol increased IL-1RA in stroke-Mo and decreased IL-8 secretion from MSCs indicating an anti-inflammatory effect of atenolol on secretomes of these cells. Captopril increased IL-8 from stroke-Mo and increased IL-6, IL-8, and MCP-1 secretions from MSCs. Captopril also increased IL-6 secretion from cocultures of stroke-Mo and MSCs indicating a strong proinflammatory effect on MSCs and their interaction with Mo. Atenolol increased the secretion of IL-8 and MCP-1 while captopril increased the secretion of IL-6 and MCP-1 from MSCs. Losartan decreased the release of IL-6 from MSCs. Losartan reduced MCP-1 and TNF-*α* from stroke-Mo and reduced IL-8 from cocultures of stroke-Mo and MSCs. Our results show that antihypertensive medications such as atenolol, captopril, and losartan, at concentrations comparable to doses prescribed for patients hospitalized for acute stroke, modulate the secretome profile of MSCs and their modulatory effects on target immune cells. Our results suggest that stroke trials involving the use of intravenous MSCs should consider the effect of these antihypertensive drugs administered to stroke patients.

## 1. Introduction

Stroke is one of the major causes of death and disability around the world. Acute stroke is characterized by a sudden increase of inflammation that leads to secondary brain injury. Cell-based therapies [1–5] are under investigation as a treatment for stroke. Among different types of cell-based therapies, human bone marrow-derived mesenchymal stromal cells (MSCs) have been shown in preclinical trials to promote recovery after stroke by releasing various biological factors called the secretome which promote immunomodulation [6, 7]. Patients with an acute ischemic stroke are prescribed medications upon admission to the hospital. Many stroke patients have comorbidities such as hypertension, have elevated blood pressure in the hospital after a stroke, and are prescribed antihypertensive medications such as beta-blockers, ACE inhibitors, and angiotensive II receptor blockers. The effects of these drugs in altering long-term outcomes after stroke have been well documented; however, the effect of these commonly prescribed drugs on MSCs is unknown. The interactions of medications with MSCs are important since clinical trials are testing the intravenous administration of these cells in stroke patients [8, 9]. Extensive studies have tested MSCs in rodent models of focal ischemic stroke where the timing of administration is 24 hrs after symptom onset. A recent meta-analysis conducted on 141 preclinical trials testing MSCs in a rodent model of ischemic stroke showed that MSCs promote functional recovery regardless of their dose, when administered up to 7 days after stroke [10]. However, clinical trials that would test the IV administration of MSCs in this time frame would involve patients being prescribed antihypertensive medications. After intravenous administration, MSCs interact with various immune cells in the circulation and peripheral organs. Among various peripheral circulating immune cells, monocytes (Mo) play an important immunoregulatory role after stroke and could be a direct target of MSCs [11, 12]. MSCs could help Mo acquire beneficial phenotypes through its secretome and hence aid in poststroke repair processes [13]. Hence, in this study, we aimed to study how antihypertensive medications change the secretomes of MSCs and the interaction of MSCs with such target immune cells as monocytes from the blood of stroke patients.

## 2. Methods

### 2.1. Isolation and Culture of Human Mesenchymal Stromal Cells (MSCs)

MSCs were isolated from commercially available fresh human bone marrow aspirates (AllCells, Alameda, CA) using density centrifugation and plastic adherence as previously described [14]. An adherent population of MSCs was obtained 3 weeks after the initiation of culture. The cells were screened for typical spindle-like morphology and growth kinetics. These MSCs strongly expressed MSC markers CD73 and CD90 and were negative for hematopoietic markers HLA-DR, CD11b, CD34, CD45, and CD19 as previously described [15]. The cells were further expanded by plating 10^6^ passage 2 cells at 200 cells/cm^2^ in 2528 cm^2^ in Nunc™ Cell Factory™ Systems with complete culture medium (CCM) that consisted of *α*-minimal essential medium (*α*-MEM; Life Technologies, Grand Island, NY), 17% fetal bovine serum (FBS; Atlanta Biologicals, Norcross, GA), 100 units/ml penicillin (Life Technologies, Carlsbad, CA), 100 *μ*g/ml streptomycin (Life Technologies, Carlsbad, CA), and 2 mM L-glutamine (Life Technologies). At 70% cell confluency, the medium was discarded, the cultures were washed with phosphate-buffered saline (PBS) (Life Technologies, Carlsbad, CA), and the adherent cells were harvested with 0.25% trypsin (Life Technologies, Carlsbad, CA) for 5 min at 37°C and frozen at 1 × 10^7^ cells/ml for subsequent experiments as passage 3.

### 2.2. Collection of Human Blood Samples

The Institutional Review Board approved all the studies and protocols involving human subjects. Peripheral blood was collected either from healthy controls or from ischemic stroke patients 24 hours after the presentation of initial symptoms through phlebotomy. Inclusion criteria for stroke patients included any acute ischemic stroke patients with NIHSS between 11 and 20.

### 2.3. Isolation of Human Peripheral Blood Monocytes

Peripheral Blood Mononuclear Cells (PBMCs) were isolated from peripheral human blood by Ficoll gradient. CD14+ monocytes (Mo) were isolated from PBMCs of healthy humans and stroke patients using an indirect magnetically labelling technique using a magnetic bead-based isolation as previously described [16]. A negative selection technique was used whereby a cocktail of biotin-conjugated monoclonal antibodies labelled nonmonocyte cells such as T cells, NK cells, B cells, dendritic cells, and basophils, and nonlabelled monocytes were collected for further cell culture work.

### 2.4. Cocultures of MSCs and Mo

Isolated monocytes were plated in a 48-well plate at 50,000 cells per well in a serum-free DMEM media. Subsequently, they were exposed to different doses of atenolol, captopril, and losartan. MSCs or an equal amount of media (control) were added in each well at 50,000 cells per well to set up a contact coculture. Monocytes exposed to drugs alone without MSCs were used as a control. After an additional 24 hours of incubation, media from monocytes exposed to drugs alone or media from contact cocultures were collected from each well and secretomes were measured. A similar method was used to collect secretomes from MSCs cultured alone in the presence of each drug.

### 2.5. Cell Proliferation Assays

MSCs were exposed to various drug concentrations of all 3 drugs. Atenolol was used from concentration ranging from 4 mM to 4 nM. Captopril was used from concentrations ranging from 5 mM to 5 nM. Losartan was used from concentrations ranging from 2 mM to 2 nM. At 24 and 48 hours of incubation, cell proliferation of MSCs was measured using MTT assay by comparing each concentration with the vehicle control.

### 2.6. Experimental Groups

MSCs as well as Mo were exposed to either atenolol (40 *μ*M to 4 nM), captopril (50 *μ*M to 5 nM), or losartan (20 *μ*M to 2 nM). The groups were as follows: (a) MSCs alone exposed to each drug, (b) healthy subject Mo alone exposed to each drug, (c) stroke patient Mo alone exposed to each drug, (d) MSC-Mo cocultures (healthy subject Mo) with each drug, and (e) MSC-Mo cocultures (stroke patient Mo) with each drug. The dose range for each drug was selected based on the plasma concentrations these drugs might attain.

### 2.7. Analysis of Secretome Using ELISA and Multiplex Cytokine Assays

Conditioned media collected from treated MSCs, Mo, and MSC-Mo cocultures were analyzed for the presence of secretomes by using the MagPix magnetic bead-based ELISA assay (Millipore) as previously described [16, 17]. Data were averaged for 3 donors. Briefly, 96-well Magpix plates were used and supernatant media were incubated with magnetic cytokine beads overnight at 4°C. The next day, detection antibodies were added and incubated for 1 hour at room temperature. A Luminex Magpix plate reader was used to measure the concentrations of multiple cytokines in the supernatant.

### 2.8. Statistical Analysis

We evaluated the dose effect of atenolol, captopril, and losartan on MSCs and Mo from healthy control or stroke patients through a mixed-effect model. We applied base-2 logarithm transformation on fold change data to normalize the secretome levels of the interested biomarkers. Mixed models were fitted to the normalized data. In a mixed model, for each source of Mo alone, MSC alone, and Mo-MSC cocultures, we considered dose level as the fixed effect. The effects from biological replicates of Mo and MSCs were considered as random effects. Based on the mixed model, we estimated log_2_(fold change) on the secretome levels for different dose levels. All analyses were performed using SAS 9.4 (Cary, NC), and a *p* value less than 0.05 was considered as significant.

## 3. Results

The following experimental groups were analyzed: (a) MSCs alone, (b) healthy subject Mo alone, (c) stroke patient Mo alone, (d) MSC-Mo cocultures with healthy subject Mo, and (e) MSC-Mo cocultures with stroke patient Mo. Each of the experimental groups was exposed to either atenolol (40 *μ*M to 4 nM), captopril (50 *μ*M to 5 nM), or losartan (20 *μ*M to 2 nM).

### 3.1. Clinically Prescribed Medications Do Not Alter the Proliferation of MSCs at Physiologically Relevant Doses

When we subjected MSCs in our experiment to various doses of atenolol (ranging from 4 mM to 4 nM), captopril (ranging from 5 mM to 5 nM), and losartan (ranging from 2 mM to 2 nM) for 24 and 48 hours, we found no significant difference in the proliferation of MSCs at physiologically relevant doses of all three drugs as compared to vehicle controls (Fig S1).

### 3.2. Antihypertensive Medications Alter the Secretome of Monocytes from Healthy Controls and Stroke Patients

We subjected Mo in our experiment to therapeutically relevant doses of atenolol, captopril, and losartan. We measured the secretome levels of IL-1RA, IL-8, IL-10, MCP-1, IFN-gamma, and TNF-alpha.

#### 3.2.1. Atenolol Increased the Secretions of IL-1RA and TNF-*α* from Stroke Patient-Derived Monocytes

**Figure 1:**
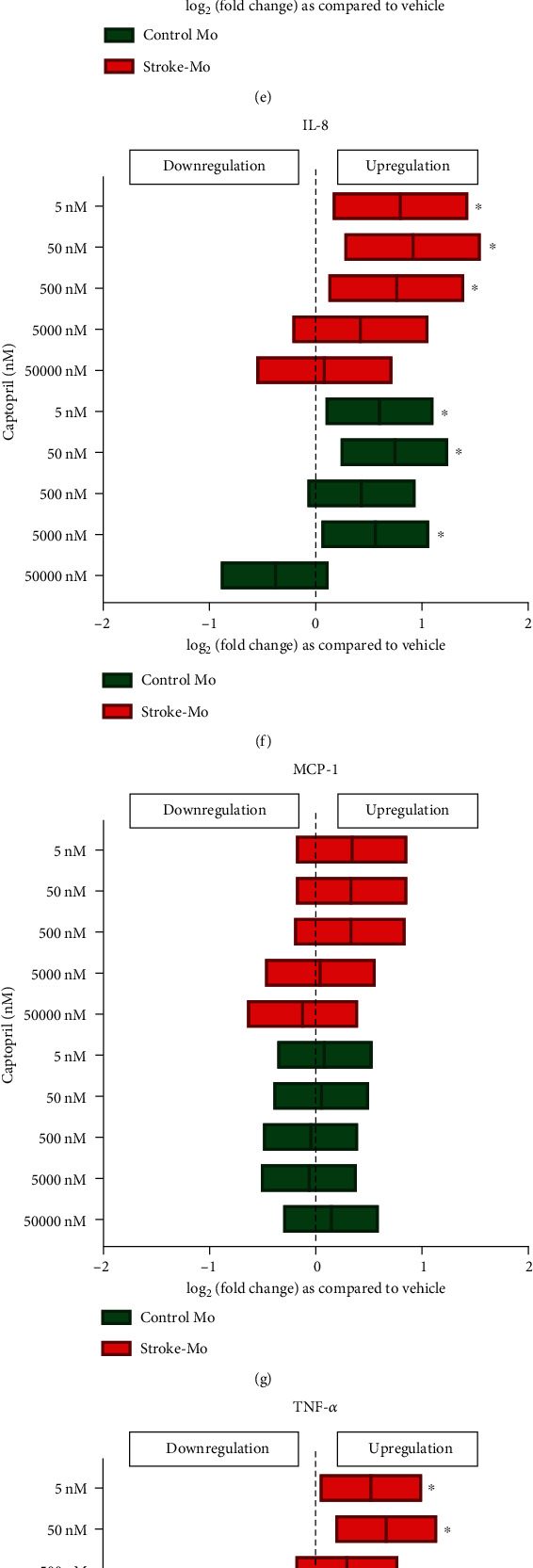
Antihypertensive medications alter the secretome of monocytes from healthy controls and stroke patients after 24 hours of exposure. Atenolol (a–d) increased IL-1RA and TNF-*α* secretions from stroke patient-derived monocytes but reduced the IL-1RA and MCP-1 secretions from health control monocytes. Captopril (e–h) increased IL-1RA secretions from stroke patient-derived monocytes but increased IL-8 secretions from both healthy control and stroke monocytes. Losartan (i–l) did not alter cytokine secretions from stroke patient-derived monocytes but reduced MCP-1 secretions from healthy control monocytes. Significance is shown by ^∗^*p* < 0.05. All fold changes are as compared to vehicle control.

Atenolol reduced the secretions of IL-1RA from Mo derived from healthy controls after 24 hours at physiologically relevant concentrations; however, it increased the secretions of IL-1RA from Mo derived from stroke patients (*p* < 0.05) (Figure 1(a)). Atenolol, at higher doses (more than 400 nM), increased the secretions of TNF-*α* from stroke-Mo (*p* < 0.05) but did not have any effect on TNF-*α* release from Mo harvested from healthy controls after 24 hours of exposure. MCP-1 secretions were reduced after 24 hours, but only from healthy control-derived monocytes and not from stroke-Mo (Figures 1(b)–1(d)).

#### 3.2.2. Captopril Increased the Secretions of IL-1RA and IL-8 from Stroke Patient-Derived Monocytes

In Mo from stroke patients, captopril after 24 hours of exposure, similar to what we have seen for atenolol, increased the secretions of IL-1RA at physiologically relevant concentrations (*p* < 0.05). Captopril also increased the secretion of IL-8 from stroke patients as well as healthy control Mo after 24 hours (*p* < 0.05). There was no effect of captopril on IL-1RA when using healthy control Mo (Figures 1(e)–1(h)).

#### 3.2.3. Losartan Did Not Have Any Effect on Secretions of IL-1RA, IL-8, MCP-1, and TNF-*α* from Stroke Patient-Derived Monocytes

Losartan at doses lower than 200 nM reduced the secretions of MCP-1 and TNF-*α* from Mo derived from healthy controls at 24 hours (*p* < 0.05); however, it did not have an effect on secretomes from stroke-Mo (Figures 1(i)–1(l)).

### 3.3. Antihypertensive Medications Alter Secretomes from MSCs

We subjected MSCs in our experiment to physiological doses of atenolol, captopril, and losartan. We measured the levels of IL-4, IL-6, IL-8, IL-10, MCP-1, IFN-gamma, and TNF-alpha. We saw significant changes only in the levels of IL-6, IL-8, and MCP-1 after MSCs were exposed to antihypertensive medications.

#### 3.3.1. Atenolol Decreases the Levels of IL-8 and MCP-1 Released from MSCs

**Figure 2:**
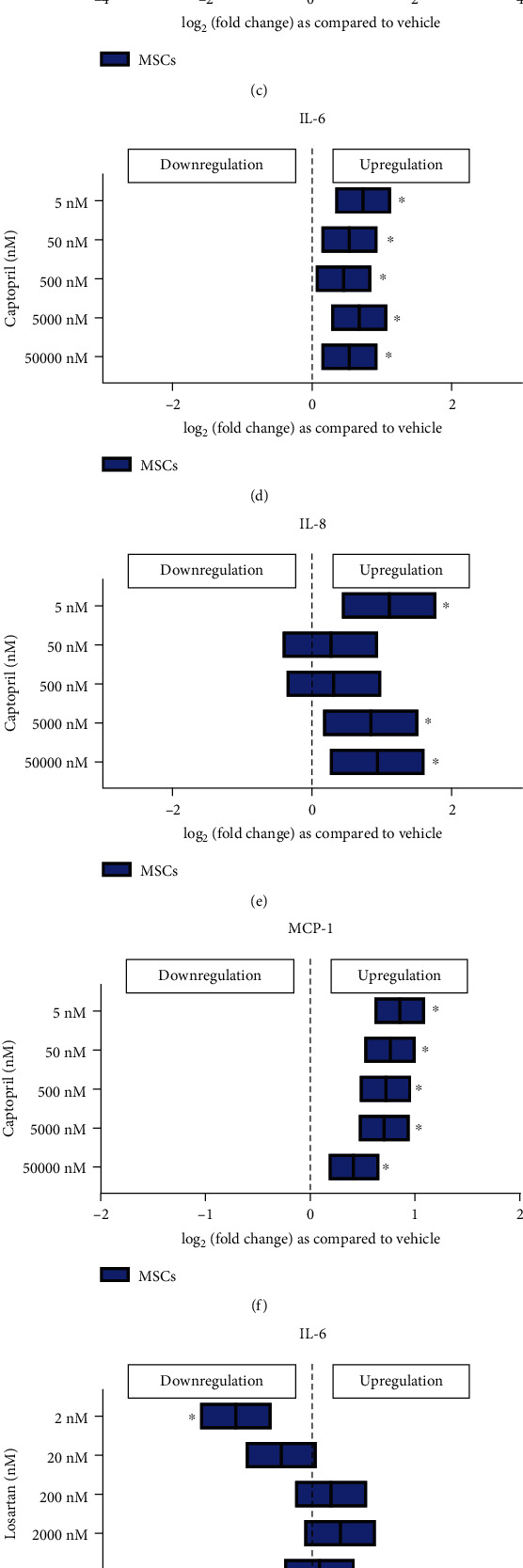
Antihypertensive medications alter the secretome of MSCs after 24 hours of exposure. Atenolol (a–c) reduced IL-6, MCP-1, and IL-8 secretions; captopril (d–f) increased IL-6, IL-8, and MCP-1 secretions; and losartan (g–i) increased IL-8 but reduced IL-6 and MCP-1 secretions. Significance is shown by ^∗^*p* < 0.05. All fold changes are as compared to vehicle control.

When MSCs were exposed to atenolol, it significantly decreased the levels of IL-8 after 24 hours of exposure. The decrease in IL-8 seen after exposure to atenolol was dose dependent, and 400 nM dose produced a significant reduction in the release of IL-8 (*p* < 0.05) (Figures 2(a)–2(c)). 40 nM and 4 nM doses produced a reduction in IL-8 secretion, but it was not significant. MCP-1 levels decreased significantly after 24 hours of exposure at 40 nM and 4 nM dose (*p* < 0.05) (Figures 2(a)–2(c)).

#### 3.3.2. Captopril Increased the Levels of IL-6, IL-8, and MCP-1 from MSCs

When MSCs were exposed to captopril, it increased the release of IL-6 significantly at all doses ranging from 500 nM to 5 nM at 24 hours after exposure (*p* < 0.05) (Figures 2(d)–2(f)). Both IL-8 and MCP-1 secretions also increased significantly from MSCs after 24 hours of exposure to captopril (*p* < 0.05) (Figures 2(d)–2(f)).

#### 3.3.3. Losartan Increased the Levels of IL-8

Losartan increased the release of IL-8 from MSCs at 24 hours at all doses except 2 nM dose at 24 hours after exposure (*p* < 0.05) (Figures 2(g)–2(i)). On the contrary, it reduced the levels of IL-6 and MCP-1 but only for the lowest dose of 2 nM at 24 hours (*p* < 0.05) (Figures 2(g)–2(i)).

### 3.4. Antihypertensive Medications Change the Secretome from MSC/Monocyte Cocultures Only when Mo Are Derived from Healthy Controls but Not from Stroke Patients

To determine whether MSCs exposed to antihypertensive medications in the presence of Mo derived from stroke patients, as compared to normal healthy controls, have different effects, we cocultured these MSCs with Mo (from stroke patients and healthy control patients) and measured the secretomes released from them.

#### 3.4.1. Atenolol Reduced the Release of Cytokines from Cocultures of MSCs with Monocytes from Healthy Controls but Had No Effect on Cocultures with Stroke Patient-Derived Monocytes

**Figure 3:**
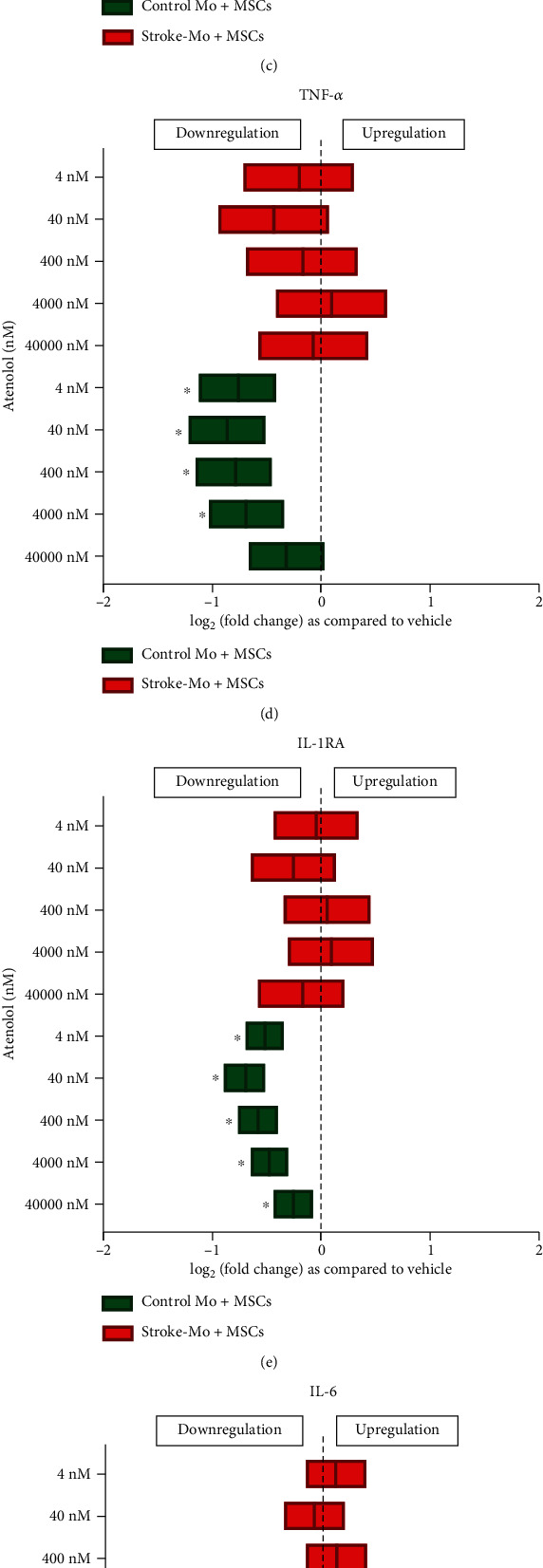
Atenolol reduces the cytokine secretions of IL-1*β*, IL-8, MCP-1, TNF-*α*, IL-IRA, IL-6, Fractalkine, and VEGF from cocultures of healthy control monocytes with MSCs after 24 hours of exposure but does not alter any cytokine secretions from cocultures involving stroke patient-derived monocytes. Significance is shown by ^∗^*p* < 0.05. All fold changes are as compared to vehicle control.

Atenolol reduced the release of IL-1*β*, IL-8, MCP-1, TNF-*α*, IL-1RA, IL-6, Fractalkine, and VEGF from cocultures of MSCs with Mo from healthy controls after 24 hours of exposure (*p* < 0.05). Atenolol had no effect on secretome in cocultures with stroke-Mo (Figure 3).

#### 3.4.2. Captopril Increased the Secretion of IL-6 from Cocultures of MSCs with Stroke Patient-Derived Mo

**Figure 4:**
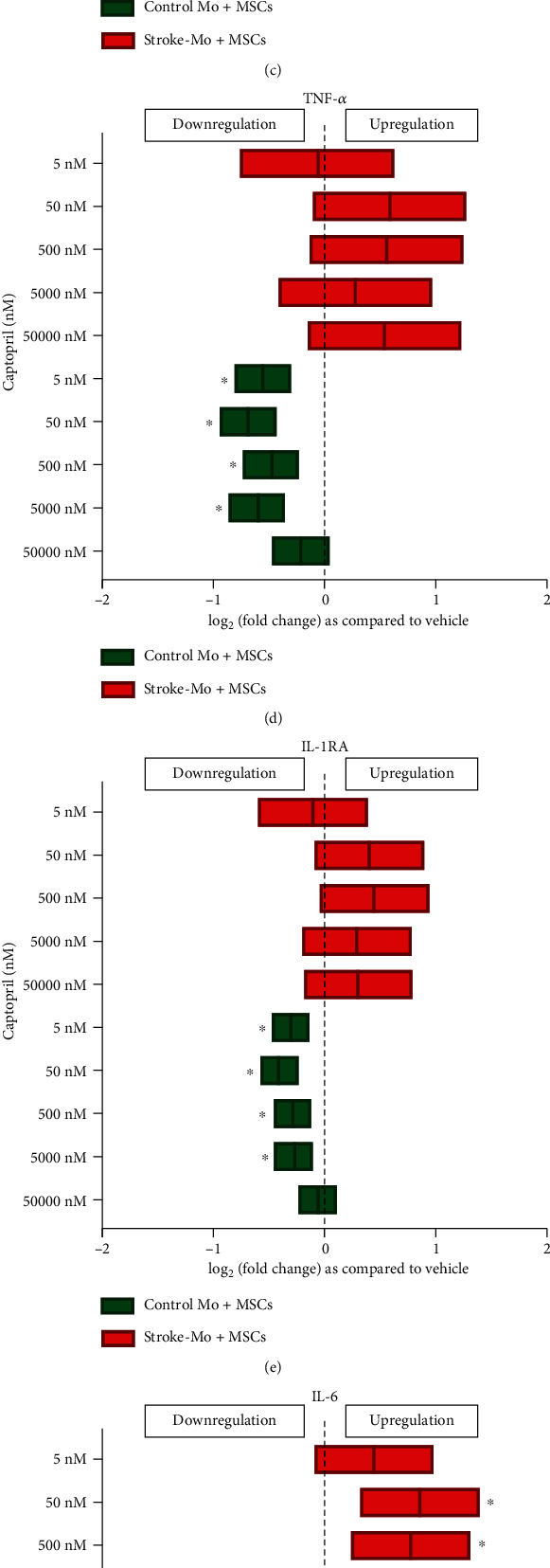
Captopril reduces the cytokine secretions of IL-8, MCP-1, TNF-*α*, IL-1RA, and IL-6 from cocultures of healthy control monocytes with MSCs after 24 hours but increases only IL-6 secretions from cocultures involving stroke patient-derived monocytes without changing any other cytokine secretions. Significance is shown by ^∗^*p* < 0.05. All fold changes are as compared to vehicle control.

Captopril reduced secretions of IL-8, MCP-1, TNF-*α*, and IL-1RA from cocultures of MSCs with healthy control monocytes but had no effect on these secretomes in cocultures with stroke-Mo after 24 hours of exposure. More importantly, captopril increased the secretions of IL-6 from cocultures of MSCs with stroke-Mo at therapeutically relevant doses at 24 hours (5000 nM to 50 nM, *p* < 0.05) (Figure 4). On the contrary, captopril exposure reduced the IL-6 secretions from cocultures of MSCs with healthy control monocytes at 24 hours of exposure (Figure 4).

#### 3.4.3. Losartan Reduced the Release of Cytokines from Cocultures of MSCs with Monocytes from Healthy Controls

**Figure 5:**
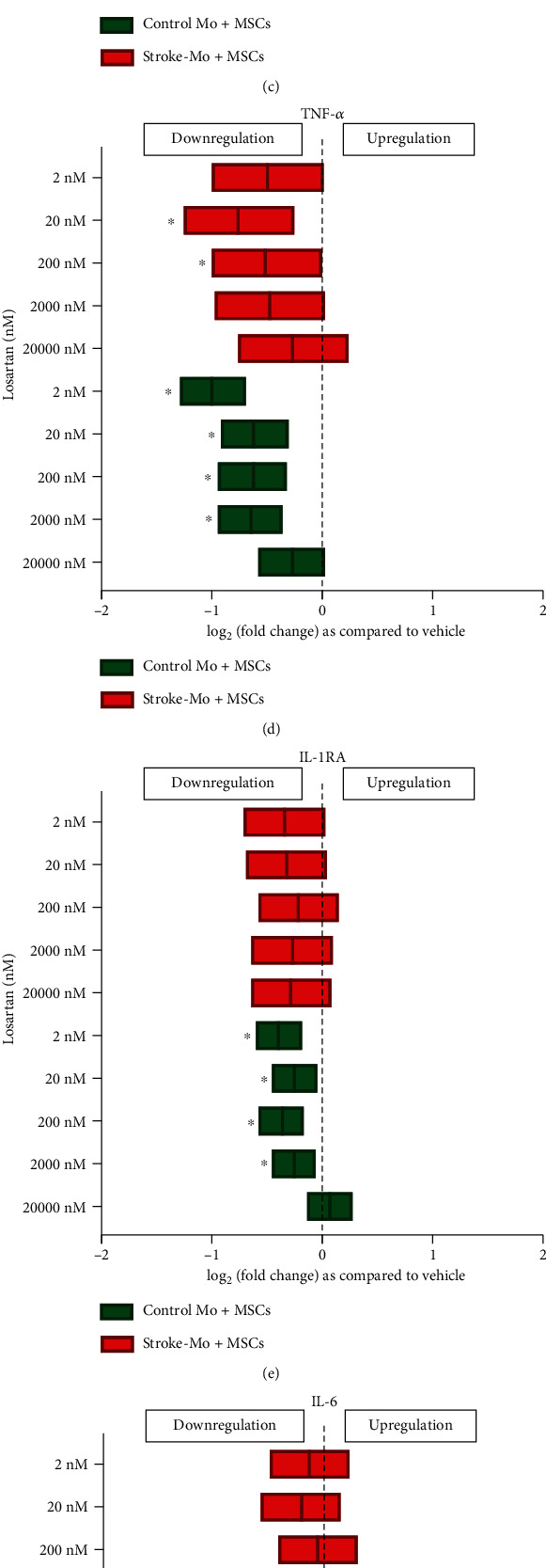
Losartan reduces the cytokine secretions of IL-1*β*, IL-8, MCP-1, TNF-*α*, IL-1RA, IL-6, and VEGF from cocultures of healthy control monocytes with MSCs after 24 hours of exposure, while decreasing only IL-8 secretions from cocultures involving stoke patient-derived monocytes without changing any other cytokine secretions. Significance is shown by ^∗^*p* < 0.05. All fold changes are as compared to vehicle control.

Losartan reduced the release of IL-8 from cocultures of MSCs with both stroke-derived and healthy control monocytes after 24 hours of exposure (Figure 5). On the contrary, for all other cytokines measured (IL-1*β*, MCP-1, TNF-*α*, IL-1RA, and IL-6), secretions were reduced only from cocultures with healthy monocytes, and not from stroke-Mo at 24 hours (Figure 5).

## 4. Discussion

The release of biological factors is considered to play an important role underlying how MSCs exert beneficial effects in stroke [18, 19]. The number of passages, storage conditions, and types of solvents of MSCs impact the viability and immunomodulatory effects of MSCs [20–24] [17]. Stroke patients also take concurrent medications for their comorbidities. A recent STEPS 4 consortium recommended the need to study the effect of these concurrent medications on cell-based therapies [25]. Since intravenously administered MSCs have advanced to clinical trials in stroke patients, we posed clinically relevant questions about the effects of antihypertensive medications commonly taken by hospitalized stroke patients because of preexisting hypertension. We sought to evaluate the effects of these medications on Mo and MSCs by specifically studying Mo- and MSC-derived secretomes and the immunomodulatory effects of MSCs on monocytes. A range of drug concentrations was studied to simulate clinically relevant drug ranges in a patient's bloodstream and to assess for dose-dependent effects. In addition, we studied secretome released from each of these cell types. IL-1*β* and TNF-*α* are known to play a proinflammatory role after stroke and worsen stroke outcomes [26, 27]. IL-8 and MCP-1 are both chemotactic factors and attract immune cells towards the brain after ischemic stroke [26]. IL-6 is a proinflammatory cytokine and plays a key role in the pathogenesis of stroke because of its ability to play a dual role [26]. Fractalkine is a proinflammatory chemokine, whose downregulation is beneficial in stroke [26]. IL-1RA and VEGF are well-known anti-inflammatory and angiogenic cytokines, respectively. Hence, we selected these cytokines to get a broader picture of immunomodulation after MSCs are exposed to atenolol, captopril, and losartan.

Atenolol is a second-generation beta-1-selective adrenergic antagonist which is indicated for the treatment of hypertension, angina pectoris, and acute myocardial infarction. For hypertension, atenolol is usually given at the dose of 50-100 mg/day [28]. After a 100 mg dose, the peak plasma concentration of atenolol reaches around 600 ng/ml after 3 hours of administration and decreases to 50-70 ng/ml after 24 hours [29]. This translates to around 2.25 *μ*M concentration in plasma at 3 hours to 200 nM concentration at 24 hours. Hence, we studied the following doses for atenolol: 40 *μ*M, 4 *μ*M, 400 nM, 40 nM, and 4 nM, to encompass the entire therapeutic range for atenolol. Atenolol increased IL-1RA release from Mo derived from stroke patients but had no significant effect on healthy control Mo at therapeutic levels. On the contrary, atenolol decreased the IL-1RA secretions from healthy control Mo, indicating that it may have different effects in stroke patients. Atenolol also reduced the secretions of IL-6, IL-8, and MCP-1 from MSCs indicating a beneficial anti-inflammatory effect on them. MCP-1 is a key chemokine that regulates the migration and infiltration of monocytes and macrophages [30]. Our results raise the possibility that atenolol could alter the release of MCP-1 from MSCs in stroke patients enrolled in an MSC trial. Reducing MCP-1 might therefore alter the effect of MSCs to promote migration of monocytes to the brain. Atenolol also reduced the secretions of IL-8 at 400 nM dose. IL-8 is known as a neutrophil chemotactic factor and has two important immunomodulatory functions. IL-8 promotes chemotaxis in target cells, primarily neutrophils, and assists their migration towards the site of injury. IL-8 also stimulates phagocytosis and is a potent promoter of angiogenesis. Reducing IL-8 can lead to negative regulation of phagocytosis and angiogenesis, thereby reducing the clearance of dead cells around the damaged stroke brain, as well as reducing the formation of new blood vessels, thereby potentially altering stroke recovery. When atenolol was exposed to cocultures of MSCs and Mo, curiously, they did not change secretions of any cytokines in cocultures when using monocytes from stroke patients. On the other hand, with cocultures involving healthy control Mo, secretions of all cytokines were reduced. In addition, when we compared the effect of atenolol between stroke patient-derived Mo alone and their cocultures with MSCs, higher doses of atenolol showed an increase in TNF-*α* secretions from stroke patient-derived Mo alone. However, this effect was abolished in the presence of MSCs, indicating the possibility that MSCs may be able to curb the TNF-*α* secretions from stroke patient-derived Mo in the presence of atenolol. Overall, atenolol did show some anti-inflammatory tendency towards Mo and MSC alone, but there was no consistent beneficial effect in cocultures unlike aspirin which has been shown to produce beneficial effects on cocultures of MSCs and stroke-derived monocytes [16].

Captopril is an angiotensin-converting enzyme (ACE) inhibitor and prevents the conversion of angiotensin I to angiotensin II. For hypertension, captopril can be given at doses ranging from 25 mg to 150 mg twice per day (BID) or three times per day (TID) [31]. The plasma concentrations of captopril range from 15 to 250 ng/ml, which translates to a concentration of 1000 nM to 50 nM [32]. To study the entire therapeutic range, we used 50 *μ*M, 5 *μ*M, 500 nM, 50 nM, and 5 nM captopril concentrations. Captopril increased IL-8 secretion from stroke as well as healthy control Mo indicating that they could have proinflammatory effects on monocytes. When MSCs were exposed to captopril, IL-6, IL-8, and MCP-1 secretions increased significantly indicating a strong inflammatory response. Cocultures with stroke-Mo and MSCs showed a significant increase in IL-6 secretion when they were exposed to captopril. There was no change in cytokine secretion for other cytokines with cocultures of stroke-Mo and MSCs, but cocultures with healthy Mo showed a consistent decrease in secretions of IL-1RA, IL-8, MCP-1, and TNF-*α*. In addition, when we compared the effect of captopril between stroke patient-derived Mo alone and their cocultures with MSCs, captopril showed an increase in IL-8 and TNF-*α* secretions from stroke patient-derived Mo alone. However, this effect was abolished in the presence of MSCs, indicating the possibility that MSCs may be able to curb the IL-8 and TNF-*α* secretions from stroke patient-derived Mo in the presence of captopril. Overall, captopril showed a consistent proinflammatory tendency towards both MSCs and stroke-Mo.

Losartan is a selective and competitive angiotensin II receptor blocker. It is approved as one of the first-line drugs for stage 1 hypertension [33]. Losartan is usually given at doses ranging from 25 to 100 mg once a day (OD). The peak serum levels of losartan after a 50 mg dose was between 200 and 250 ng/ml. However, serum levels can range from 50 to 300 ng/ml with 100 ng OD dosage [34]. This translates to a serum concentration range of 200 nM to 1200 nM. In this study, we used 20 *μ*M, 2 *μ*M, 200 nM, 20 nM, and 2 nM concentrations of losartan. Losartan did not change secretions of any measured cytokines from stroke-derived Mo but reduced MCP-1 and TNF-*α* from healthy control Mo. When MSCs were exposed to losartan, there was a consistent increase in IL-8 secretion. On the contrary, IL-8 secretion was consistently decreased in cocultures of MSCs and Mo for both stroke-Mo and healthy control Mo. Cocultures of MSCs and stroke-Mo failed to show any significant change of other released cytokines, but cocultures involving healthy Mo showed consistently reduced secretions of all cytokines measured in this study. Losartan did not show any differences in secretome when stroke patient-derived Mo alone was compared with cocultures of stroke-Mo and MSCs, indicating that losartan does not change the effects of MSCs on stroke-Mo.

ACE inhibitors or ARBs are commonly given to stroke patients to control blood pressure. MSCs exposed to the ACE inhibitor, captopril (doses ranging from 5000 nM to 5 nM), showed increased secretion of IL-6, IL-8, and MCP-1 at 24 hours after exposure. MSCs exposed to the ARB, losartan, showed a significant reduction in IL-6 secretion at 2 nM dose but showed a significant increase in IL-8 secretion at all doses higher than 20 nM. Our results indicate that blocking ACE enhances proinflammatory signals from MSCs while blocking angiotensin II receptor reduces proinflammatory signals from MSCs. A study by Krikov et al. showed that blocking angiotensin II receptor reduced stroke volume and improved functional outcome significantly, while blocking ACE did not produce any such effect [35]. Our results are consistent with this effect and in fact take it one step further by indicating that MSC treatment could be beneficial in combination with losartan by reducing IL-6 at low doses but not captopril, which increases IL-6 secretions. Our results also show that there is no consistent effect of these drugs on cocultures of MSCs and stroke-derived Mo, even though the secretome was markedly reduced from cocultures with healthy control-derived Mo. The only significant change was with captopril, which increased IL-6 secretion from cocultures involving stroke-Mo but reduced IL-6 from cocultures with healthy Mo. The limitation of this study is that it is difficult to attribute secretome change due to these drugs to either Mo or MSCs in the coculture experiments. However, in a clinical scenario involving MSCs in stroke patients, both Mo and MSCs will be present. Antihypertensive drugs are a variable which will change from patient-to-patient. Hence, our study provides a framework for designing clinical trials involving MSCs in stroke patients on these medications. Our results also strongly indicate that atenolol may have an anti-inflammatory effect on the secretome when administered in stroke patients, while captopril could be proinflammatory for secretomes derived from interactions between MSCs and Mo.

Overall, our results show that antihypertensive medications at clinically relevant doses have significant effects on the secretomes and immunomodulatory signaling of MSCs. Since immunomodulation is a key mechanism of MSCs in promoting stroke recovery in animal studies, our results suggest the possibility that antihypertensive medications may exert drug interactions on MSCs and exposure to these medications may be an important variable that should be considered in clinical trials testing MSCs in stroke patients.

## Data Availability

The data used to support the findings of this study are available from the corresponding author upon request.

## Supplementary Materials

Supplementary materials Supplementary Figure S1: supplementary figure showing that the clinically relevant drugs do not alter the cell proliferation of MSCs at physiologically relevant concentrations after 24 and 48 hours of exposure to atenolol, captopril, and losartan.
