# Supplementary material for: Medications for Hypertension Change the Secretome Profile from Marrow Stromal Cells and Peripheral Blood Monocytes
Source: Stem Cells Int. 2020 Aug 1;2020:8894168. doi: 10.1155/2020/8894168 (PMC7416264; doi:10.1155/2020/8894168)

Supplementary Material

Medications for hypertension change the secretome profile from marrow stromal cells and peripheral blood monocytes

Nikunj Satani[1#], Kaavya Giridhar[1], Chunyan Cai[2], Natalia Wewior[1], Dominique D. Norris[1], Jaroslaw Aronowski[1], Sean I. Savitz[1]

[1]Institute for Stroke and Cerebrovascular Diseases, McGovern Medical School at UTHealth, Houston, Texas, USA
[2]Center for Clinical and Translational Sciences, McGovern Medical School at UTHealth, Houston, Texas, USA

[#]Corresponding Author:
Nikunj Satani, MD, MPH
Institute of Stroke and Cerebrovascular Diseases
McGovern Medical School at UTHealth
6431 Fannin Street, MSB 7.628, Houston TX 77030
Phone: 713-500-5512
Email: Nikunj.B.Satani@uth.tmc.edu

Figure S1. Clinically relevant drugs does not alter cell proliferation of MSCs at physiological concentrations. MTT assay was performed after 24 and 48 hours of exposure to Atenolol **(A)**, Captopril **(B)** or Losartan **(C)**. Absorbance was measured at 595nm. Data is normalized with vehicle control for that drug.

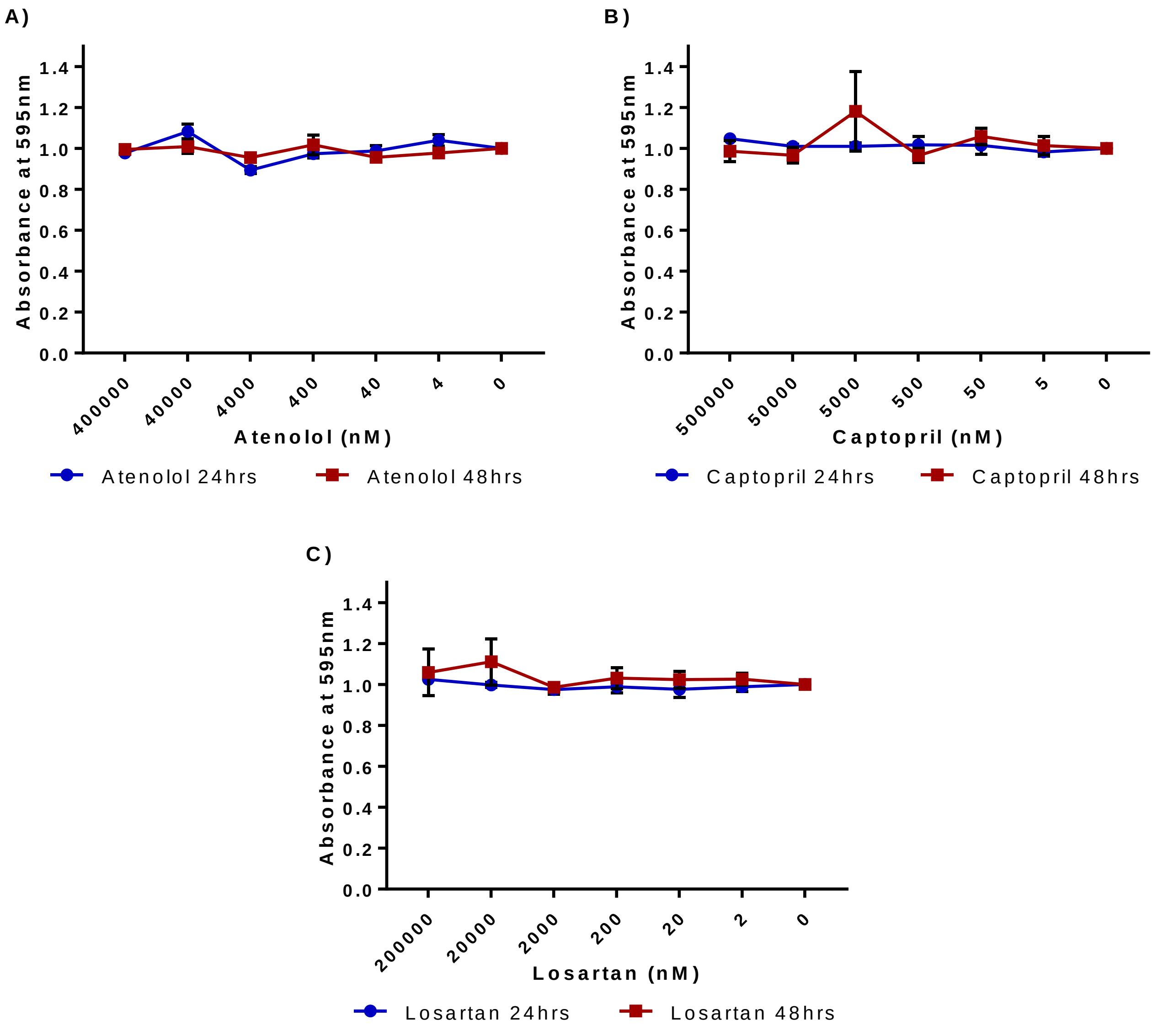

Supplement: Supplementary materials — Supplementary Figure S1: supplementary figure showing that the clinically relevant drugs do not alter the cell proliferation of MSCs at physiologically relevant concentrations after 24 and 48 hours of exposure to atenolol, captopril, and losartan. [file 8894168.f1.pdf]
